# Genetic hypervariability of a Northeastern Atlantic venomous rockfish

**DOI:** 10.7717/peerj.11730

**Published:** 2021-07-12

**Authors:** Sara M. Francisco, Rita Castilho, Cristina S. Lima, Frederico Almada, Francisca Rodrigues, Radek Šanda, Jasna Vukić, Anna Maria Pappalardo, Venera Ferrito, Joana I. Robalo

**Affiliations:** 1 MARE—Marine and Environmental Sciences Centre, ISPA Instituto Universitário de Ciências Psicológicas, Sociais e da Vida, Lisbon, Portugal; 2 Centro de Ciências do Mar (CCMAR), Universidade do Algarve, Faro, Portugal; 3 Department of Zoology, National Museum, Prague, Czeck Republic; 4 Faculty of Science, Department of Ecology, Charles University, Prague, Czech Republic; 5 Department of Biological, Geological and Environmental Sciences, Section of Animal Biology Biology ‘‘Marcello La Greca’’, University of Catania, Catania, Italy

**Keywords:** Population structure, Connectivity, Atlantic-Mediterranean transition, Range expansion, *Scorpaena maderensis*, Scorpaenidae

## Abstract

**Background:**

Understanding the interplay between climate and current and historical factors shaping genetic diversity is pivotal to infer changes in marine species range and communities’ composition. A phylogeographical break between the Atlantic and the Mediterranean has been documented for several marine organisms, translating into limited dispersal between the two basins.

**Methods:**

In this study, we screened the intraspecific diversity of 150 individuals of the Madeira rockfish (*Scorpaena maderensis*) across its distributional range (seven sampling locations in the Atlantic and Mediterranean basins) using the mitochondrial control region and the nuclear S7 first intron.

**Results:**

The present work is the most comprehensive study done for this species, yielding no genetic structure across sampled locations and no detectable Atlantic-Mediterranean break in connectivity. Our results reveal deep and hyper-diverse bush-like genealogies with large numbers of singletons and very few shared haplotypes. The genetic hyper-diversity found for the Madeira rockfish is relatively uncommon in rocky coastal species, whose dispersal capability is limited by local oceanographic patterns. The effect of climate warming on the distribution of the species is discussed.

## Introduction

Understanding the interplay between historical and current climate changes in shaping genetic diversity is pivotal to infer future changes in the distribution of marine species. Climate warming is expected to significantly impact marine organisms with range disturbances anticipated (e.g., Cheung et al., 2009; Lasram et al., 2010; Hollowed et al., 2013; Poloczanska et al., 2014; Lenoire & Svenning, 2015; García Molinos et al., 2016). Indeed, climate-induced contractions, expansions, shifts and population extirpations across distribution areas have been amply recorded (e.g., Nicastro et al., 2013; Yeruham et al., 2015; Yu & Chen, 2018; Aguilera et al., 2020). In the past three decades, several advances in molecular and analytical tools led to the accumulation of data on the genetic diversity of marine organisms (e.g., Helyar et al., 2011; Plough, 2016; Allendorf, 2017) and connectivity (for reviews see Selkoe et al., 2016; Bryan-Brown et al., 2017). Different phylogeographic patterns have been recorded in the North-eastern Atlantic, ranging from panmictic species with homogeneous genetic diversity throughout their distribution range to species with marked population differentiation (e.g., Francisco et al., 2011; Jenkins, Castilho & Stevens, 2018).

Traditionally, the degree of genetic connectivity has been related to contemporary and historical factors that combined shape the present-day observed patterns (e.g., Gaggiotti et al., 2009; Woodall, Koldewey & Shaw, 2011; Reis-Santos et al., 2018). Marine species’ geographical spread and population differentiation may be influenced by their potential for dispersal and gene flow, depending on passive larval dispersal and active adult migratory movements (e.g., Selkoe & Toonen, 2011; Pascual et al., 2017). The interaction between life-history traits such as pelagic larval duration (PLD), larval behaviour and swimming abilities, post-settlement processes and oceanographic regimes play a role in the dispersal range of species with low adult dispersal ability (e.g., Bowen et al., 2006; Galarza et al., 2009; Schunter et al., 2011; Dalongeville et al., 2015) (see Bryan-Brown et al. (2017) for a review). However, there is still an ongoing debate on the reliability of using a species’ PLD as a proxy for population connectivity (e.g., Bradbury et al., 2008; Weersing & Toonen, 2009).

Amongst the historical factors shaping present-day connectivity patterns are climate changes associated with the Pleistocene glacial cycles, geological barriers, salinity gradients, hydrodynamics and paleoecological history (e.g., Olsen et al., 2004; Bigg et al., 2008; Palero et al., 2008). These very dynamic factors are recurrently invoked to explain the biogeography of marine organisms (e.g., Jenkins, Castilho & Stevens, 2018). At the peak of the last glacial maximum (LGM), around 21 thousand years ago (kya), the sea level dropped by 110–150 m (Lambeck & Purcell, 2005). Still, the connection between the Mediterranean and the adjacent Atlantic waters was not interrupted (Flores et al., 1997), sustaining glacial refugia hypothesised within this basin (Maggs et al., 2008).

The Atlantic-Mediterranean phylogeographic break is well documented for several marine organisms (e.g., Magoulas et al., 2006; Taboada & Pérez-Portela, 2016), notwithstanding the reopening of the Gibraltar Strait connection at the end of the Messinian Salinity Crisis (~5.33 Mya) (Hsü, Ryan & Cita, 1973; Krijgsman et al., 1999; Duggen et al., 2003). For others, often closely related species, the gene flow is unconstrained (e.g., Stamatis et al., 2004; Castilho et al., 2017; Lourenço et al., 2017) (see Patarnello, Volckaert & Castilho, 2007 and Kettle et al., 2011 for reviews). For the former, the inferred barrier has been associated with hydrological conditions that prevent migration either in recent times or in the Pleistocene, or both. This soft barrier location, however, is not consistent and for some taxa, the observed phylogeographic break is at the Strait of Gibraltar in the entrance of the Mediterranean basin (e.g., Sala-Bozano, Ketmaier & Mariani, 2009; Fruciano et al., 2011; García-Merchán et al., 2012), whilst for several others is further East, at the Almeria-Oran front in the Alboran Sea region (e.g., Zane et al., 2000; Lemaire, Versini & Bonhomme, 2005; Viñas et al., 2014).

One of the evident effects of climate change is the increase in seawater temperature, which translates into a global tropicalization trend (e.g., Bianchi & Morri, 2003; Wernberg et al., 2016) that also affects the Northeastern Atlantic. Along the southern and western coasts of the Iberian Peninsula, several organisms are expanding their poleward distribution, some with great impact in community composition (e.g., Lourenço et al., 2012; Nicastro et al., 2013; Bode et al., 2020; Robalo et al., 2020). One of the fish species recently reported off southwestern continental Portugal is the Madeira rockfish, *Scorpaena maderensis* Valenciennes 1833 (Encarnação et al., 2019), an estimated moderately vulnerable species (36/100) according to the model by Cheung, Pitcher & Pauly (2005). The IUCN Red List highlights the unknown current population trend of this least concerned species (Nunoo et al., 2015). Its genetic characterization and phylogeography have therefore become imperative.

The Madeira rockfish, *Scorpaena maderensis*, is distributed in the eastern Atlantic, including the islands of Azores, Madeira, Canaries and Cape Verde and in the Mediterranean Sea (Hureau & Litvinenko, 1986; Eschmeyer et al., 1990; Froese & Pauly, 2019). The species is a benthic sedentary species, mostly occupying shallow coastal areas with rocky bottoms and estuaries (usually underneath boulders or in crevices). Congeneric species, *S. scrofa* and *S. porcus*, have high residency and narrow home ranges (Özgül et al., 2019). Based on the present-day known distribution, mainly of subtropical nature, the estimated seawater temperature range for the species is 16–25 °C (Kaschner et al., 2019), but there are no studies on the thermal tolerance of the species. The Madeira rockfish is a generalized and opportunistic feeder of benthic or epibenthic crustaceans and, occasionally, algae, gastropods, polychaetes and small fishes (La Mesa, La Mesa & Tomassetti, 2007), and shows sexual dimorphism in growth rate, maximum size and longevity, with differences registered between the Mediterranean and Azorean populations (Morato et al., 2001; La Mesa, La Mesa & Micalizzi, 2005). A specialized mode of oviparity is described for the genus and the eggs are deposited as a whole in a protective gelatinous matrix that facilitates spawning cohesiveness and floatation (Wourms & Lombardi, 1992). The spawning season takes place from December to February in the Mediterranean (La Mesa, La Mesa & Micalizzi, 2005) and from March to June in the Azores (Costa, 2007). However, structural features of its biology are yet to be clarified, particularly the ones related to reproduction and early-life traits.

The present work is the first population study for this species, comprising a wide sampling coverage of its distribution range (seven locations from the Atlantic and the Mediterranean Sea), and two molecular markers (mitochondrial and nuclear) to screen the genetic diversity of *S. maderensis* with the following objectives: (1) evaluate the genetic diversity within and among locations; (2) assess the population genetic structure of the Madeira rockfish; and (3) evaluate the putative existence of a soft barrier between the Atlantic and the Mediterranean populations.

## Materials and Methods

### Sampling

**Figure 1:**
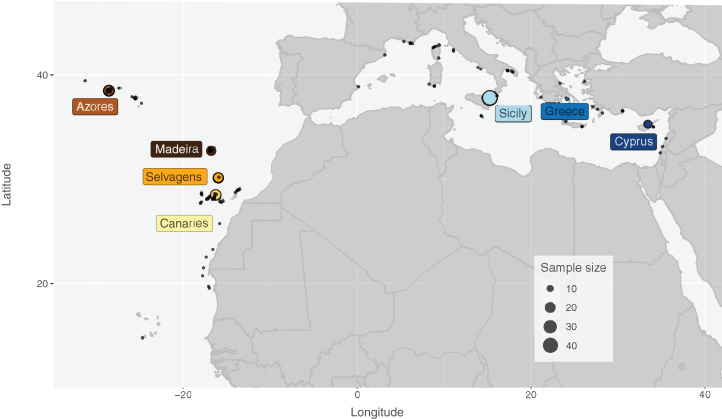
Distribution map and sampling locations for *Scorpaena maderensis*. Distribution map and sampling locations for *Scorpaena maderensis*. Black dots represent GBIF Occurrence Download (GBIF.org, 22 April 2021; https://doi.org/10.15468/dl.bpf9s8). Coloured circles represent samples used in this study and are consistent in all figures. Size of circles proportional to sample size.

Specimens of *S. maderensis* were collected from seven locations across its distributional range in the Atlantic and Mediterranean: Cyprus (CYP), Greece (GRE—Euboea), Sicily (SIC—Messina, Riposto and Siracusa; Italy), Azores (AZO—Faial; Portugal), Madeira (MAD—Funchal; Portugal), Selvagens (SEL; Portugal) and Canaries (CAN—Tenerife; Spain) (Fig.1 and Table 1). Specimens were provided by fishers as the species is a frequent by-catch in coastal short-range artisanal fisheries and fins were clipped after assessing the species identification for each individual. Samples were preserved in 96% ethanol and deposited in ISPA-IU/MARE tissue collection.

**Table 1:** Sample locations, coordinates, sizes and summary statistics for the mitochondrial control region and the nuclear S7 gene of *Scorpaena maderensis*.

Location	Code	Long	Lat	Mitochondrial Control Region									Nuclear S7								
				*N*	*n*_H_	*n*_P_	*n*_S_	*n*_P/H_	*h*	π (%)	*R*	*pR*	*N*	*n*_H_	*n*_P_	*n*_S_	*n*_P/H_	*h*	π (%)	*R*	*pR*
Cyprus	CYP	35.091	33.344	13	12	9	11	75.00%	0.987	2.12	9.733	6.166	22	22	19	22	86.36%	1.000	3.05	19.209	9.714
Greece	GRE	37.740	24.059	7	7	7	7	100.00%	1.000	3.66	7.000	5.503	14	12	10	10	83.33%	0.978	3.42	12.000	5.553
Sicily	SIC>	37.708	15.202	42	31	19	22	61.29%	0.984	2.50	11.741	5.780	22	22	20	22	90.91%	1.000	2.93	19.209	10.032
*Mediterranean*	MED	–	–	62	47	37	36	78.72%	0.989	2.58	12.445	3.872	58	54	50	50	92.59%	0.998	3.05	24.002	2.130
Canaries	CAN	28.47	−16.255	22	19	11	16	57.89%	0.987	2.77	11.037	6.053	44	40	32	37	80.00%	0.995	3.05	22.381	15.128
Selvagens	SEL	30.108	−15.957	20	20	15	20	75.00%	1.000	3.02	11.667	8.097	48	47	39	46	82.98%	0.999	2.92	23.775	16.675
Madeira	MAD	32.694	−16.775	20	20	17	20	85.00%	1.000	3.28	11.667	8.629	32	27	18	22	66.67%	0.990	3.13	19.662	10.923
Azores	AZO	38.522	−28716	22	19	12	16	63.16%	0.987	2.79	11.037	4.215	38	32	25	28	78.13%	0.989	3.04	20.502	12.814
*Atlantic*	ATL	–	–	84	68	58	57	85.29%	0.993	3.01	12.897	4.215	162	127	123	113	96.85%	0.992	2.87	24.752	8.478
*Total*	–	–	–	146	105	–	81	–	0.989	2.87	–	–	220	177	–	159	–	0.995	3.60	–	–

**Note:**

Long, longitude; Lat, latitude; *N*, number of gene copies; *n_H_*, number of haplotypes; *n_P_*, number of private haplotypes; *n_S_*, singletons; *n_P/H_*, proportion of private haplotypes; *h*, haplotype diversity; *π*, nucleotide diversity; *R*, allelic richness; *pR*, private allelic richness.

### DNA extraction, amplification and sequencing

Total genomic DNA was extracted with the REDExtract-N-Amp Kit (Sigma-Aldrich, St. Louis, MO, USA) following the manufacturer’s instructions. The mitochondrial control region (CR) and the first intron of the nuclear S7 ribosomal protein gene (S7) were amplified, in a Bio-Rad Mycycler thermal cycler, using primers L-pro1 and H-DL1 (Ostellari et al., 1996), and S7RPEX1F and S7RPEX2R (Chow & Hazama, 1998). The PCR protocol was performed in a 20 μl total reaction volume with 10 μl of REDExtract-N-ampl PCR mix (Sigma-Aldrich, St. Louis, MO, USA), 0.8 μl of each primer (10 μM), 4.4 μl of Sigma water and 4 μl of template DNA using the following PCR conditions: initial denaturation at 94 °C for 7′, followed by 35/30 cycles (denaturation at 94 °C for 30/45″, annealing at 55 °C for 30/45″, and extension at 72 °C for 1′; values CR/S7, respectively) and a final extension at 72 °C for 7′. The forward primers (L-pro1 and S7RPEX1F) were used for the sequencing reaction, and the PCR products were purified and sequenced in STABVIDA (http://www.stabvida.net/).

Chromatograms were manually checked, edited with Codon Code Aligner (Codon Code Corporation, http://www.codoncode.com/index.htm) and sequences were aligned with Clustal X 2.1 (Larkin et al., 2007). For S7, chromatograms were checked for double peaks (see Fig. S1 in Supplemental Materials) and, whenever possible, both strands of the same specimen were recovered following the approach of Sousa-Santos et al. (2005). All sequences were deposited in GenBank (Accession numbers MN716857–MN717002; and MN717003–MN717124, respectively for CR and S7) (Table S1 in Supplementary Materials).

### Molecular data analyses

The genetic diversity and population structure of *S. maderensis* were assessed using several packages developed for R v.4.0.2 (R Core Team, 2020), in RStudio (RStudio Team, 2020). We used haplotypes (Aktas, 2020) and pegas (Paradis, 2010) R-packages to estimate standard descriptive measures of genetic diversity, including number of haplotypes and private haplotypes, haplotype diversity (*h*, Nei, 1987) and nucleotide diversity (π, Nei, 1987) and respective standard deviations. The software HP-Rare (Kalinowski, 2005) was used to estimate allelic richness (*R*) and private allelic richness (*pR*), using rarefaction to correct for sample-size bias associated with the relative abundance or easiness to collect samples of this species. For the S7 gene fragment the programme ARLEQUIN v3.5 (Excoffier & Lischer, 2010) was used to reconstruct the haplotypes with the ELB algorithm (Excoffier, Laval & Balding, 2003), and to perform the exact probability tests for deviations from the Hardy–Weinberg equilibrium (HWE) (Guo & Thompson, 1992). The same software was used to assess population structure, performing analyses of molecular variance (AMOVA) (Excoffier, Smouse & Quattro, 1992). The *diveRsity* R-package (Keenan et al., 2013) was used to evaluate the genetic structure, estimating fixation (*F*_*ST*_ (Weir & Cockerham, 1984), Nei’s *G*_*ST*_ (Nei, 1987), Hedrick’s *G’*_*ST*_ (Hedrick, 2005)) and allelic differentiation (Jost’s *D* (Jost, 2009)) measures. For both fragments, the PopART software (Leigh & Bryant, 2015) was used to build TCS haplotype networks (Clement, Posada & Crandall, 2000) based on the parsimony methodology by Templeton, Crandall & Sing (1992).

## Results

For the CR, a fragment of 354 bp was amplified and the 146 sequences obtained defined 105 haplotypes, with a total of 80 polymorphic sites found. Differences among haplotypes corresponded to 80 transitions, 12 transversions and 1 indel. For the S7 the 220 sequences (110 individuals) defined a total of 177 haplotypes. For this marker, the fragment obtained was 517 bp long and the differences among haplotypes corresponded to 206 mutations (70 transitions, 80 transversions and 50 indels). The *S. maderensis* S7 dataset, as a whole, conformed to the HWE (*p* = 0.998), although 36 out of the 171 polymorphic sites were in heterozygote deficit. For both fragments, the genetic diversity indices were generally very high, with little variation among collection sites (Table 1). The proportion of private haplotypes was high for all the locations (Table 1), with only 9.52% and 2.26% being shared between the Atlantic and the Mediterranean, for the CR and the S7, respectively.

**Figure 2:**
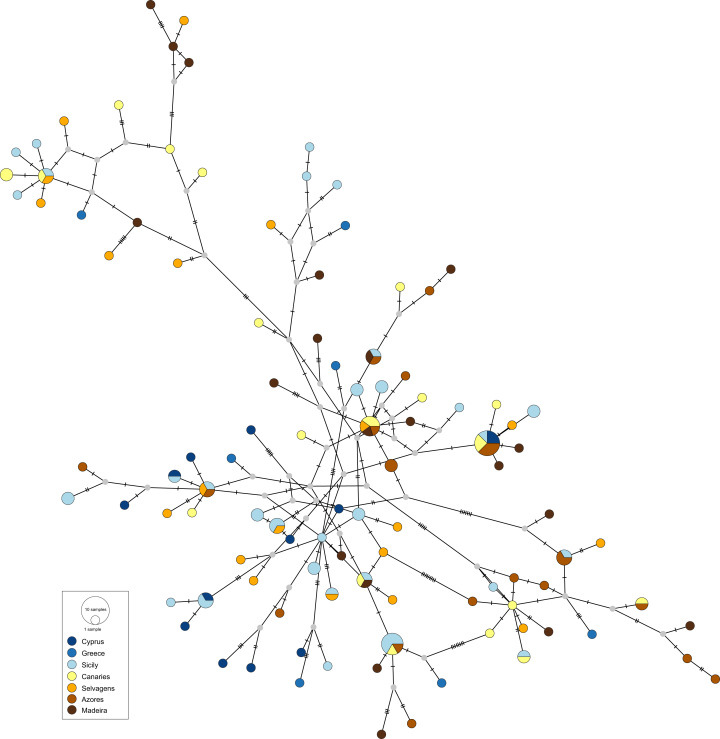
Haplotype network for the mitochondrial control region of *Scorpaena maderensis*. Colours refer to sampling locations (the same as in Fig. 1). The area of the circles is proportional to haplotype frequencies. In the case where haplotypes are shared among sampling locations, the colours represented are proportional to the frequency of the haplotype in each sampling location.

**Figure 3:**
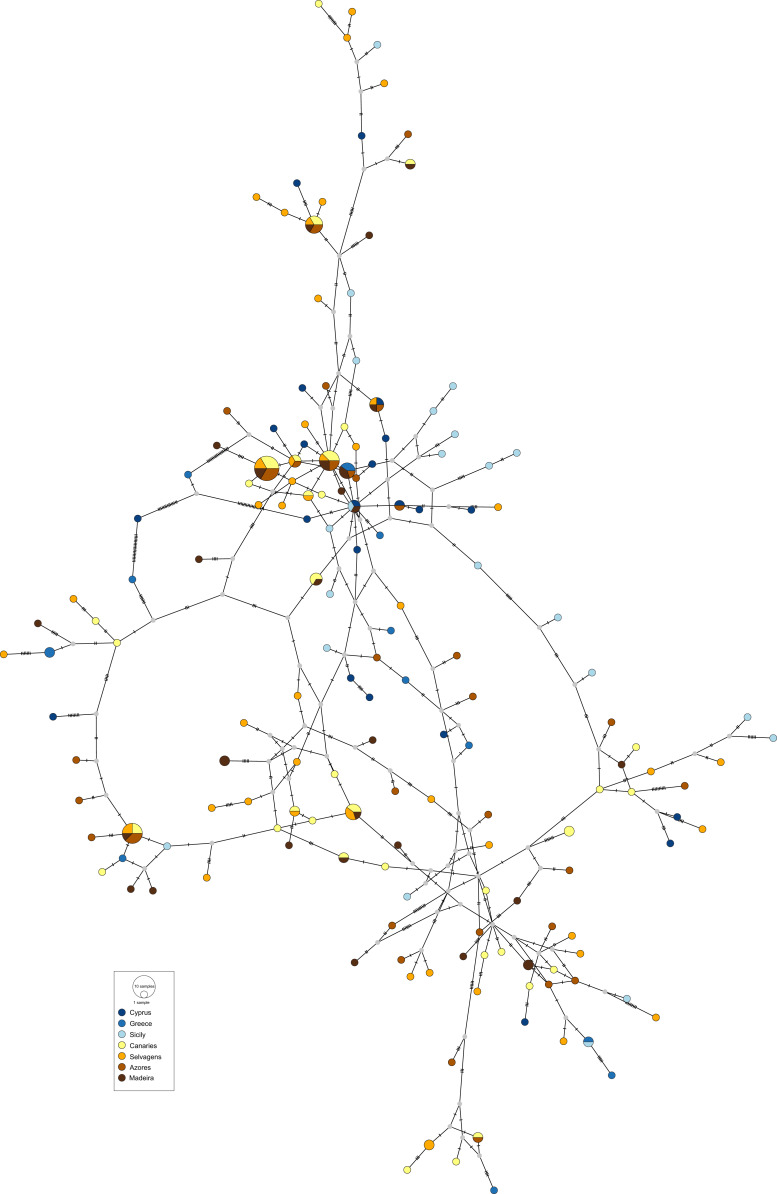
Haplotype network for the nuclear S7 gene of *Scorpaena maderensis*. Colours refer to sampling locations (the same as in Fig. 1). The area of the circles is proportional to haplotype frequencies. In the case where haplotypes are shared among sampling locations, the colours represented are proportional to the frequency of the haplotype in each sampling location

The obtained haplotype networks revealed deep hyper-diverse bush-like genealogies, with a large number of singletons, few shared haplotypes and no evidence for geographic structure (CR: Fig. 2, S7: Fig. 3; see also Table 1, details on haplotype composition are given in Table S1 in Supplementals Materials).

The divergence parameters yielded significant values for the overall CR (Table 2). In both markers, results from the pairwise comparisons were equivocal, with *F*_*ST*_ showing non-significant values, Nei’s *G*_*ST*_ revealing significant values for some of the comparisons, and Hedrick’s *G’*_*ST*_ and Jost’s *D* yielding all comparisons statistically significant (Table 2), i.e., all pairs of sampling sites usually have distinct haplotypes. In fact, eight (CR and S7) out of 22 pairwise comparisons revealed complete haplotypic differentiation (*D* = 1), including between some of the geographically closest sampling sites (Table 2). This high structuring was supported by the AMOVA results (CR: *F*_*ST*_ = 0.031, *p* = 0.004; S7: *F*_*ST*_ = 0.016, *p* = 0.005), which also revealed that variation among sampling sites accounted for only 3.08% (CR) and 1.58% (S7) of the total variation (Table S2 in Supplemental Materials).

**Table 2:** Pairwise differentiation for the mitochondrial CR and nuclear S7 of *Scorpaena maderensis*.

Pairwise	*F*_*ST*_	95% CI	*G*_*ST*_	95% CI	*G′*_*ST*_	95% CI	*D*	95% CI
*Mitochondrial control region*								
CYP-GRE	0.007	[0.000–0.098]	0.033	[−0.003 to 0.089]	**1.000**	[1.000–1.000]	**1.000**	[1.000–1.000]
CYP-SIC>	0.006	[0.000–0.098]	0.016	[0.000–0.039]	**0.775**	[0.574–0.878]	**0.771**	[0.564–0.879]
CYP-CAN	0.000	[0.000–0.044]	0.016	[−0.002 to 0.040]	**0.688**	[0.390–0.816]	**0.683**	[0.375–0.816]
CYP-SEL	0.006	[0.000–0.050]	**0.020**	[0.004–0.045]	**1.000**	[1.000–1.000]	**1.000**	[1.000–1.000]
CYP-MAD	0.006	[0.000–0.049]	**0.020**	[0.004–0.044]	**1.000**	[1.000–1.000]	**1.000**	[1.000–1.000]
CYP-AZO	0.000	[0.000–0.043]	0.016	[−0.002 to 0.040]	**0.688**	[0.385–0.815]	**0.683**	[0.371–0.816]
GRE-SIC>	0.009	[0.000–0.080]	0.027	[−0.001 to 0.076]	**1.000**	[1.000–1.000]	**1.000**	[1.000–1.000]
GRE-CAN	0.007	[0.000–0.084]	0.029	[−0.001 to 0.080]	**1.000**	[1.000–1.000]	**1.000**	[1.000–1.000]
GRE-SEL	0.000	[0.000–0.050]	0.026	[−0.003 to 0.076]	**1.000**	[1.000–1.000]	**1.000**	[1.000–1.000]
GRE-MAD	0.000	[0.000–0.049]	0.026	[−0.004 to 0.076]	**1.000**	[1.000–1.000]	**1.000**	[1.000–1.000]
GRE-AZO	0.007	[0.000–0.083]	0.029	[−0.001 to 0.079]	**1.000**	[1.000–1.000]	**1.000**	[1.000–1.000]
SIC-CAN	0.005	[0.000–0.030]	**0.011**	[0.001–0.025]	**0.699**	[0.472–0.845]	**0.696**	[0.465–0.846]
SIC-SEL	0.004	[0.000–0.025]	**0.011**	[0.002–0.023]	**0.825**	[0.671–0.901]	**0.823**	[0.666–0.901]
SIC-MAD	0.007	[0.000–0.028]	**0.013**	[0.005–0.024]	**0.956**	[0.864–0.975]	**0.956**	[0.861–0.975]
SIC-AZO	0.004	[0.000–0.029]	**0.011**	[0.001–0.024]	**0.666**	[0.390–0.816]	**0.662**	[0.431–0.815]
CAN-SEL	0.000	[0.000–0.029]	0.012	[0.000–0.028]	**0.781**	[0.558–0.868]	**0.779**	[0.549–0.868]
CAN-MAD	0.000	[0.000–0.029]	0.012	[0.000–0.028]	**0.781**	[0.556–0.869]	**0.779**	[0.548–0.869]
CAN-AZO	0.000	[0.000–0.028]	0.010	[−0.004 to 0.027]	**0.545**	[0.254–0.743]	**0.540**	[0.243–0.744]
SEL-MAD	0.000	[0.000–0.024]	**0.012**	[0.001–0.026]	**0.904**	[0.749–0.938]	**0.903**	[0.743–0.938]
SEL-AZO	0.002	[0.000–0.030]	**0.013**	[0.002–0.028]	**0.854**	[0.678–0.913]	**0.852**	[0.671–0.913]
MAD-AZO	0.002	[0.000–0.030]	**0.013**	[0.002–0.028]	**0.854**	[0.675–0.913]	**0.852**	[0.668–0.913]
MED-ATL	0.003	[0.000–0.010]	**0.005**	[0.002–0.009]	**0.615**	[0.446–0.747]	**0.613**	[0.443–0.747]
*Nuclear S7*								
CYP-GRE	0.034	[0.000–0.052]	**0.021**	[0.006–0.044]	**1.000**	[1.000–1.000]	**1.000**	[1.000–1.000]
CYP-SIC>	0.022	[0.000–0.020]	**0.011**	[0.002–0.022]	**0.913**	[0.764–0.942]	**0.911**	[0.759–0.942]
CYP-CAN	0.017	[0.000–0.019]	**0.010**	[0.004–0.019]	**1.000**	[1.000–1.000]	**1.000**	[1.000–1.000]
CYP-SEL	0.014	[0.000–0.015]	**0.008**	[0.002–0.017]	**0.946**	[0.854–0.964]	**0.945**	[0.852–0.964]
CYP-MAD	0.020	[0.000–0.022]	**0.011**	[0.003–0.022]	**0.887**	[0.746–0.932]	**0.884**	[0.741–0.932]
CYP-AZO	0.019	[0.000–0.022]	**0.011**	[0.003–0.021]	**0.902**	[0.777–0.941]	**0.900**	[0.773–0.941]
GRE-SIC>	0.032	[0.000–0.051]	**0.019**	[0.003–0.043]	**0.923**	[0.764–0.955]	**0.920**	[0.756–0.955]
GRE-CAN	0.022	[0.000–0.052]	**0.019**	[0.006–0.042]	**1.000**	[1.000–1.000]	**1.000**	[1.000–1.000]
GRE-SEL	0.020	[0.000–0.049]	**0.018**	[0.004–0.040]	**1.000**	[1.000–1.000]	**1.000**	[1.000–1.000]
GRE-MAD	0.025	[0.000–0.046]	**0.017**	[0.002–0.039]	**0.794**	[0.560–0.885]	**0.787**	[0.549–0.885]
GRE-AZO	0.026	[0.000–0.056]	**0.021**	[0.007–0.044]	**1.000**	[1.000–1.000]	**1.000**	[1.000–1.000]
SIC-CAN	0.017	[0.000–0.200]	**0.010**	[0.004–0.019]	**1.000**	[1.000–1.000]	**1.000**	[1.000–1.000]
SIC-SEL	0.015	[0.011–0.016]	**0.009**	[0.003–0.017]	**1.000**	[1.000–1.000]	**1.000**	[1.000–1.000]
SIC-MAD	0.021	[0.000–0.023]	**0.012**	[0.004–0.022]	**0.943**	[0.832–0.966]	**0.942**	[0.828–0.966]
SIC-AZO	0.020	[0.000–0.024]	**0.012**	[0.005–0.022]	**1.000**	[1.000–1.000]	**1.000**	[1.000–1.000]
CAN-SEL	0.010	[0.000–0.007]	0.004	[0.000–0.009]	**0.599**	[0.403–0.729]	**0.596**	[0.399–0.729]
CAN-MAD	0.013	[0.000–0.013]	0.006	[0.000–0.014]	**0.571**	[0.340–0.727]	**0.565**	[0.335–0.728]
CAN-AZO	0.011	[0.000–0.011]	0.005	[0.000–0.012]	**0.513**	[0.278–0.686]	**0.507**	[0.273–0.686]
SEL-MAD	0.012	[0.000–0.013]	**0.006**	[0.001–0.013]	**0.689**	[0.496–0.804]	**0.685**	[0.491–0.804]
SEL-AZO	0.011	[0.000–0.010]	0.005	[0.000–0.011]	**0.547**	[0.335–0.702]	**0.542**	[0.331–0.703]
MAD-AZO	0.013	[0.000–0.015]	0.006	[−0.001 to 0.015]	**0.484**	[0.239–0.668]	**0.477**	[0.234–0.669]
MED-ATL	0.006	[0.000–0.009]	**0.005**	[0.003–0.008]	**0.904**	[0.829–0.947]	**0.903**	[0.828–0.947]

**Note:**

*F_ST_*, Nei’s *G_ST_*, Hedrick’s *G’_ST_* and Jost’s *D*. Significant values in bold (95% confidence interval not overlapping with zero).

## Discussion

The present work comprises a wide geographic sampling coverage of *Scorpaena maderensis* with locations from the Atlantic and the Mediterranean Sea and a molecular dataset with two markers. Our results highlight two main features in the population genetics of the Madeira rockfish: (1) deep hyper-diverse bush-like genealogies, characterised by large numbers of singletons and few shared haplotypes; and (2) absence of genetic structure across sampled locations, with no detectable Atlantic-Mediterranean break in connectivity. Before discussing these findings in detail, we address the main caveats concerning this study: the sampling strategy and the molecular markers used. Although most locations are represented by numbers of individuals in line with previous phylogeographic studies in marine species, one can a posteriori posit that the high number of singleton haplotypes found is biased by insufficient sampling. In fact, a recent study published by our team recorded even higher genetic diversity in a coastal fish species, revealing that it would be necessary to sample a total of 700 individuals for the sampling to be representative of the population (Robalo et al., 2020). Additionally, we have no samples from intermediate locations between the Atlantic archipelagos and the Western Mediterranean. These areas are not in the reported distribution of the species (Froese & Pauly, 2019) and the few reported individuals are occasional appearances (Encarnação et al., 2019). Another caveat is using only one mitochondrial and one nuclear marker in a day and age where next-generation sequencing producing thousands of markers are being increasingly used. This study is in line with previous research in the pursuit for patterns and processes involved in the phylogeography of the species from the North-East Atlantic (e.g., Bargelloni et al., 2005; Debes, Zachos & Hanel, 2008; Francisco et al., 2011; Robalo et al., 2013a). These previous studies used the same set of markers, allowing across species comparisons and multi-species approaches (e.g., Robalo et al., 2012; Robalo et al., 2013b; Francisco et al., 2014; Almada et al., 2017; Castilho et al., 2017) while revealing very distinct patterns.

### Genetic hyper-diversity of the Madeira rockfish

All sampling locations show high diversity values, mainly due to a large proportion of singletons. There are two equally possible explanations for this result: (1) if numerous suitable temperature pockets harboured a large enough number of individuals, no demographic bottlenecks would affect the genetic composition and a high genetic variability could be maintained; (2) the species exhibit a patchy distribution near the Macaronesian islands and in scattered locations in the Mediterranean, where self-recruitment may be more dominant than larval drifting to further locations. There are instances where successive self-recruitment generations lead counter-intuitively to the maintenance of high genetic diversity (e.g., Feng, Williams & Place, 2017; Fourdrilis & Backeljau, 2019; Francisco & Robalo, 2020; Robalo et al., 2020). Furthermore, the CR sequence hypervariability may alternatively or concomitantly be explained by the mutation rate of the fragment, the evolutionary-rates hypothesis, or the metabolic rate theory as discussed in Robalo et al. (2020).

### Genetic structure of the Madeira rockfish

The present results reveal no evidence for genetic structure, geographically associated or not, and therefore we posit that the Madeira rockfish is not composed of discernible groups within the Atlantic and the Mediterranean, nor these two basins are clearly differentiated. This hypothesis is strongly dependent on the genetic markers used in the study. In studies with other species, the CR region has yielded equivocal results regarding the detection of genetic structure. We can find in the literature examples of findings of hypervariability and absence of genetic structure (e.g., Francisco et al., 2011; Mehraban et al., 2020; Song et al., 2020), and studies presenting hypervariability and significant genetic structure (e.g., Cunha et al., 2014; Castilho et al., 2017; Robalo et al., 2020). North-Eastern Atlantic past recolonization processes and historical and present dispersal movements are influenced by species-specific life-history traits, favourable oceanographic conditions, such as sea surface temperatures, and suitable recruitment habitat (e.g., Wares & Cunningham, 2001; Pappalardo et al., 2015).

The results also do not reveal any phylogeographic break between the Atlantic and the Mediterranean locations for *S. maderensis* (Fig. 2, Table 2), similarly to what has been previously recorded in other species (e.g., *Trachurus trachurus* (Comesaña, Martínez-Areal & Sanjuan, 2008), *Diplodus sargus* (Stefanni et al., 2015) and *Dentex dentex* (Viret et al., 2018)). Although many factors can explain this outcome, there are two biological characteristics that may play a relevant role: large mean pelagic larval duration and high adult dispersal capability, features common to all these species. The observed discrepancy across statistics can be attributed to their different nature (Bird et al., 2011). In cases where the geographic distribution of haplotypes is uncorrelated with the relationship among alleles, which is *S. maderensis* case (Figs. 2 and 3), the fixation indices will not accurately depict the structure, and the differences found among the different measures can often be uninformative to the underlying biology of population structuring.

The mean pelagic larval duration (PLD) influences on a certain degree a species dispersal potential before reaching the juvenile stages. Although it is recognized the PLD is not a universal driver of range size and therefore a promoter of connectivity in many fish (e.g., Weersing & Toonen, 2009; Selkoe & Toonen, 2011), in certain situations it seems to have some influence (Lester & Ruttenberg, 2005). To our knowledge there is no no data on the PLD of *S. maderensis*, but congenerics are known to spend 29 (*S. porcus* in Macpherson & Raventos (2006)) and 30 days (*S. guttata* in Carr & Reed (1993)) in the plankton, which is not a short duration. The hydrographic regime in this stretch of the North-East Atlantic is dominated by the Azores Current and its south-eastward branch, the Canary Current, a complex system of eddies (Stramma, 1984; Hernández-Guerra et al., 2001). At the Atlantic-Mediterranean transition, the eastward flowing Atlantic water describes a quasi-permanent anticyclonic gyre (Millot, 1999). The PLD and the circulation regime of the area would thus contribute to the unconstrained gene flow between the two basins and among the Macaronesian archipelagos.

Adult rockfish of the genus *Scorpaena* display a low active dispersal capacity (Özgül et al., 2019). Nevertheless, adults of the Madeira rockfish may perform short-distance movements. Short dispersal movements following suitable habitat may have happened, in the past decade, with individuals of this species being recorded for the first time in the Gorringe seamount (Abecasis et al., 2009) and in South Portugal (Encarnação et al., 2019), near the entrance of the Gibraltar Strait. Although a certain degree of connectivity is expected from the results of this study it would be interesting to investigate the origin of these newcomers, mainly because adult dispersal is one of the essential life-history patterns influencing connectivity and population structure (e.g., Francisco, Pereira & Robalo, 2019).

In conclusion, although no specific information is available regarding *S. maderensis*, its putative life-history patterns (i.e., dispersal mostly through the larval stage given the more sedentary nature of adults) is conducive to the lack of genetic structure. This lack in structure is shared by other fish groups in the same geographical areas, like gobids. A recent work on *Gobius cruentatus* (Čekovská et al., 2020, for additional species see references within), a species with a similar life-history pattern, has also revealed high genetic variability and no geographic structure with an estimated migration route following the main currents of the distribution area.

A meta-analysis to tackle whether or not climate change influences marine ecological phenomena found that over 80% of all observations were coherent with the expected impacts of climate change. Moreover, the rates of geographic distribution shifts were, on average, consistent with those needed to track ocean surface temperature changes (Poloczanska et al., 2013). It is expected that *S. maderensis* will similarly follow a trajectory compatible with its optimal physiological temperature, and therefore it may extend its geographic distribution towards north. *S. maderensis* is a species with both a commercial and a biotechnological interest, it would be of importance to conduct fishery census to detect the arrival of this species to new locations.

## Supplemental Information

10.7717/peerj.11730/supp-1 Supplemental Information 1 Detailed information for the specimens of *Scorpaena maderensis*. Detailed information for the specimens of *Scorpaena maderensis*: sampling location, specimen, GenBank Accession numbers and haplotypes for the Mitochondrial control region (CR) and the first intron of the nuclear S7 ribosomal protein gene (S7). For the S7 dataset the haplotypes given were reconstructed using the ELB algorithm.

10.7717/peerj.11730/supp-2 Supplemental Information 2 Analysis of molecular variance (AMOVA) among and within sampling locations for the mitochondrial CR and nuclear S7 of *Scorpaena maderensis*.

10.7717/peerj.11730/supp-3 Supplemental Information 3 Examples of sequencing chromatograms for the nuclear S7 gene of *Scorpaena maderensis*.

10.7717/peerj.11730/supp-4 Supplemental Information 4 Dataset with the aligned sequences of the mitochondrial control region of *Scorpaena maderensis*.

10.7717/peerj.11730/supp-5 Supplemental Information 5 Dataset with the aligned sequences of the first intron of the nuclear S7 ribosomal protein gene of *Scorpaena maderensis*.

## Additional Information and Declarations

### DNA Deposition

The following information was supplied regarding the deposition of DNA sequences: All sequences are available at GenBank: MN716857 - MN717002 and MN717003 - MN717124.

### Data Availability

The following information was supplied regarding data availability: The FASTA files with the alignments for CR and S7 markers are available as Supplemental Files.
