# Supplementary material for: Genetic hypervariability of a Northeastern Atlantic venomous rockfish
Source: PeerJ. 2021 Jul 12;9:e11730. doi: 10.7717/peerj.11730 (PMC8280884; doi:10.7717/peerj.11730)
Supplement: Supplemental Information 1 — Detailed information for the specimens of Scorpaena maderensis: sampling location, specimen, GenBank Accession numbers and haplotypes for the Mitochondrial control region (CR) and the first intron of the nuclear S7 ribosomal protein gene (S7). For the S7 dataset the haplotypes given were reconstructed using the ELB algorithm. [file peerj-09-11730-s001.docx]

| **Specimen** | **Sampling location** | **CR** | | **S7** | | |
| --- | --- | --- | --- | --- | --- | --- |
|  |  | **Accession number** | **Haplotype** | **Accession number(s)** | **Haplotypes (ELB reconstruction)** | |
| CYPJ1938 | Cyprus | MN716864 | 35 | MN717011 | 113 | 114 |
| CYPJ2029 | Cyprus | MN716865 | 36 | MN717012 | 115 | 116 |
| CYPJ2047 | Cyprus | MN716866 | 37 | MN717014-5 | 117 | 118 |
| CYPJ2048 | Cyprus | MN716867 | 1 | MN717013 | 119 | 120 |
| CYPJ2049 | Cyprus | MN716868 | 38 | MN717016 | 121 | 122 |
| CYPJ2065 | Cyprus | MN716869 | 39 | MN717017 | 123 | 124 |
| CYPJ2066 | Cyprus | MN716870 | 1 | MN717018 | 16 | 125 |
| CYPJ2067 | Cyprus | MN716871 | 40 | - | - | - |
| CYPJ2121 | Cyprus | MN716872 | 41 | MN717019 | 126 | 127 |
| CYPJ2122 | Cyprus | MN716873 | 42 | MN717020 | 15 | 128 |
| CYPJ2123 | Cyprus | MN716874 | 43 | MN717021 | 129 | 130 |
| CYPJ2124 | Cyprus | MN716875 | 44 | MN717022 | 10 | 131 |
| CYPJ2125 | Cyprus | MN716876 | 45 | - | - | - |
| GREC2292 | Greece | MN716857 | 46 | MN717003-4 | 132 | 133 |
| GREC2293 | Greece | MN716858 | 47 | MN717005 | 134 | 21 |
| GREC2302 | Greece | MN716859 | 48 | MN717006 | 135 | 136 |
| GREC2303 | Greece | MN716860 | 49 | MN717007 | 137 | 138 |
| GREC2304 | Greece | MN716861 | 50 | MN717008 | 2 | 2 |
| GRED3004 | Greece | MN716862 | 51 | MN717009 | 139 | 18 |
| GRED3005 | Greece | MN716863 | 52 | MN717010 | 140 | 18 |
| SICME1 | Sicily | MN716961 | 44 | - | - | - |
| SICME2 | Sicily | MN716962 | 6 | - | - | - |
| SICME3 | Sicily | MN716963 | 1 | - | - | - |
| SICME4 | Sicily | MN716964 | 41 | - | - | - |
| SICME5 | Sicily | MN716965 | 55 | - | - | - |
| SICME6 | Sicily | MN716966 | 56 | - | - | - |
| SICME7 | Sicily | MN716967 | 57 | MN717114 | 141 | 142 |
| SICME8 | Sicily | MN716968 | 58 | - | - | - |
| SICME9 | Sicily | MN716969 | 56 | MN717115 | 143 | 144 |
| SICME10 | Sicily | MN716970 | 53 | MN717116 | 145 | 146 |
| SICME11 | Sicily | MN716971 | 6 | - | - | - |
| SICME12 | Sicily | MN716972 | 2 | - | - | - |
| SICME13 | Sicily | MN716973 | 31 | - | - | - |
| SICME14 | Sicily | MN716974 | 6 | - | - | - |
| SICME15 | Sicily | MN716975 | 54 | - | - | - |
| SICRI3 | Sicily | MN716976 | 6 | MN717117 | 147 | 148 |
| SICRI4 | Sicily | MN716977 | 63 | MN717118 | 149 | 150 |
| SICRI5 | Sicily | MN716978 | 64 | MN717119 | 151 | 152 |
| SICRI6 | Sicily | MN716979 | 61 | MN717120 | 153 | 154 |
| SICRI7 | Sicily | MN716980 | 60 | MN717121 | 155 | 156 |
| SICRI8 | Sicily | MN716981 | 62 | MN717122 | 17 | 157 |
| SICRI9 | Sicily | MN716982 | 41 | - | - | - |
| SICRI10 | Sicily | MN716983 | 59 | MN717123 | 158 | 159 |
| SICRI11 | Sicily | MN716984 | 26 | MN717124 | 22 | 160 |
| SICRI12 | Sicily | MN716985 | 60 | - | - | - |
| SICRI13 | Sicily | MN716986 | 61 | - | - | - |
| SICRI14 | Sicily | MN716987 | 59 | - | - | - |
| SICRI15 | Sicily | MN716988 | 62 | - | - | - |
| SICSR2 | Sicily | MN716989 | 9 | - | - | - |
| SICSR3 | Sicily | MN716990 | 70 | - | - | - |
| SICSR4 | Sicily | MN716991 | 14 | - | - | - |
| SICSR5 | Sicily | MN716992 | 71 | - | - | - |
| SICSR6 | Sicily | MN716993 | 72 | - | - | - |
| SICSR7 | Sicily | MN716994 | 34 | - | - | - |
| SICSR8 | Sicily | MN716995 | 69 | - | - | - |
| SICSR9 | Sicily | MN716996 | 73 | - | - | - |
| SICSR10 | Sicily | MN716997 | 65 | - | - | - |
| SICSR11 | Sicily | MN716998 | 66 | - | - | - |
| SICSR12 | Sicily | MN716999 | 67 | - | - | - |
| SICSR13 | Sicily | MN717000 | 68 | - | - | - |
| SICSR14 | Sicily | MN717001 | 69 | - | - | - |
| SICSR15 | Sicily | MN717002 | 67 | - | - | - |
| CAN01 | Canaries | MN716899 | 10 | MN717044 | 22 | 23 |
| CAN02 | Canaries | - | - | MN717045 | 24 | 4 |
| CAN03 | Canaries | MN716900 | 1 | MN717046 | 11 | 13 |
| CAN04 | Canaries | MN716901 | 17 | MN717047 | 15 | 25 |
| CAN05 | Canaries | MN716902 | 20 | MN717048 | 11 | 16 |
| CAN06 | Canaries | MN716903 | 6 | MN717049 | 26 | 8 |
| CAN07 | Canaries | MN716904 | 21 | MN717050 | 5 | 161 |
| CAN08 | Canaries | MN716905 | 22 | MN717051 | 27 | 19 |
| CAN09 | Canaries | MN716906 | 23 | MN717052 | 19 | 1 |
| CAN10 | Canaries | MN716907 | 24 | MN717053 | 12 | 28 |
| CAN11 | Canaries | MN716908 | 25 | MN717054 | 29 | 30 |
| CAN12 | Canaries | MN716909 | 1 | MN717055-6 | 13 | 31 |
| CAN13 | Canaries | MN716910 | 26 | MN717057 | 32 | 6 |
| CAN14 | Canaries | MN716911 | 27 | MN717058 | 8 | 11 |
| CAN15 | Canaries | MN716912 | 28 | MN717059 | 33 | 5 |
| CAN16 | Canaries | MN716913 | 29 | MN717060 | 34 | 12 |
| CAN17 | Canaries | MN716914 | 30 | MN717061 | 35 | 36 |
| CAN18 | Canaries | - | - | MN717062 | 1 | 37 |
| CAN19 | Canaries | - | - | MN717063 | 38 | 12 |
| CAN20 | Canaries | MN716915 | 31 | MN717064-5 | 39 | 40 |
| CAN21 | Canaries | MN716916 | 32 | MN717066 | 8 | 41 |
| CAN22 | Canaries | MN716917 | 33 | MN717067 | 11 | 42 |
| CAN24 | Canaries | MN716918 | 34 | - | - | - |
| CAN28 | Canaries | MN716919 | 22 | - | - | - |
| CAN29 | Canaries | MN716920 | 17 | - | - | - |
| MAD01 | Madeira | MN716941 | 76 | MN717095 | 3 | 99 |
| MAD02 | Madeira | MN716942 | 86 | MN717096 | 100 | 101 |
| MAD03 | Madeira | MN716943 | 31 | - | - | - |
| MAD04 | Madeira | MN716944 | 88 | - | - | - |
| MAD05 | Madeira | MN716945 | 89 | - | - | - |
| MAD06 | Madeira | MN716946 | 90 | MN717097 | 16 | 102 |
| MAD07 | Madeira | MN716947 | 74 | MN717098 | 103 | 104 |
| MAD08 | Madeira | MN716948 | 75 | MN717099-100 | 105 | 106 |
| MAD09 | Madeira | MN716949 | 17 | MN717101 | 17 | 12 |
| MAD10 | Madeira | MN716950 | 77 | MN717102 | 5 | 20 |
| MAD11 | Madeira | MN716951 | 78 | MN717103-4 | 107 | 108 |
| MAD12 | Madeira | MN716952 | 79 | MN717105 | 9 | 8 |
| MAD13 | Madeira | MN716953 | 80 | MN717106 | 109 | 10 |
| MAD14 | Madeira | MN716954 | 2 | MN717107 | 11 | 18 |
| MAD15 | Madeira | MN716955 | 81 | MN717108 | 20 | 11 |
| MAD16 | Madeira | MN716956 | 82 | MN717109 | 18 | 12 |
| MAD17 | Madeira | MN716957 | 83 | MN717110 | 3 | 110 |
| MAD18 | Madeira | MN716958 | 84 | - | - | - |
| MAD19 | Madeira | MN716959 | 85 | MN717111 | 6 | 21 |
| MAD20 | Madeira | MN716960 | 87 | MN717112-3 | 111 | 112 |
| SEL01 | Selvagens | MN716921 | 34 | MN717068 | 43 | 5 |
| SEL02 | Selvagens | MN716922 | 91 | MN717069 | 44 | 45 |
| SEL03 | Selvagens | MN716923 | 92 | MN717070 | 46 | 47 |
| SEL04 | Selvagens | MN716924 | 93 | MN717071 | 48 | 6 |
| SEL05 | Selvagens | MN716925 | 94 | MN717072-3 | 14 | 49 |
| SEL06 | Selvagens | MN716926 | 95 | MN717074-5 | 50 | 51 |
| SEL07 | Selvagens | MN716927 | 96 | MN717076 | 52 | 53 |
| SEL08 | Selvagens | MN716928 | 97 | MN717077 | 54 | 55 |
| SEL09 | Selvagens | MN716929 | 64 | MN717078 | 6 | 56 |
| SEL10 | Selvagens | MN716930 | 98 | MN717079 | 57 | 58 |
| SEL11 | Selvagens | MN716931 | 105 | MN717080 | 12 | 59 |
| SEL12 | Selvagens | MN716932 | 14 | MN717081-2 | 60 | 61 |
| SEL14 | Selvagens | - | - | MN717083 | 11 | 13 |
| SEL15 | Selvagens | MN716933 | 99 | MN717084 | 62 | 63 |
| SEL16 | Selvagens | MN716934 | 100 | MN717085 | 64 | 65 |
| SEL17 | Selvagens | MN716935 | 101 | MN717086 | 7 | 6 |
| SEL18 | Selvagens | MN716936 | 102 | MN717087 | 10 | 11 |
| SEL19 | Selvagens | MN716937 | 103 | MN717088 | 8 | 66 |
| SEL20 | Selvagens | MN716938 | 66 | MN717089 | 67 | 68 |
| SEL21 | Selvagens | MN716939 | 17 | MN717090 | 69 | 70 |
| SEL22 | Selvagens | - | - | MN717091 | 71 | 5 |
| SEL23 | Selvagens | - | - | MN717092 | 72 | 4 |
| SEL24 | Selvagens | - | - | MN717093 | 73 | 74 |
| SEL25 | Selvagens | MN716940 | 104 | MN717094 | 75 | 76 |
| AZO01 | Azores | MN716877 | 1 | MN717023 | 77 | 11 |
| AZO02 | Azores | MN716878 | 15 | MN717024 | 97 | 11 |
| AZO03 | Azores | MN716879 | 19 | MN717025 | 78 | 79 |
| AZO04 | Azores | MN716880 | 11 | MN717026 | 17 | 80 |
| AZO05 | Azores | MN716881 | 2 | MN717027 | 81 | 10 |
| AZO06 | Azores | MN716882 | 1 | MN717028 | 11 | 82 |
| AZO07 | Azores | MN716883 | 3 | - | - | - |
| AZO08 | Azores | MN716884 | 4 | MN717029 | 14 | 12 |
| AZO09 | Azores | MN716885 | 5 | MN717030 | 7 | 11 |
| AZO10 | Azores | MN716886 | 6 | MN717031 | 83 | 84 |
| AZO11 | Azores | MN716887 | 7 | MN717032 | 8 | 9 |
| AZO12 | Azores | MN716888 | 8 | MN717033 | 85 | 86 |
| AZO13 | Azores | MN716889 | 9 | MN717034 | 87 | 88 |
| AZO14 | Azores | MN716890 | 10 | MN717035 | 5 | 89 |
| AZO15 | Azores | MN716891 | 9 | MN717036 | 90 | 91 |
| AZO16 | Azores | MN716892 | 11 | MN717037 | 18 | 92 |
| AZO17 | Azores | MN716893 | 12 | MN717038-9 | 93 | 94 |
| AZO18 | Azores | MN716894 | 13 | MN717040-1 | 95 | 96 |
| AZO19 | Azores | MN716895 | 14 | MN717042 | 5 | 5 |
| AZO20 | Azores | MN716896 | 16 | MN717043 | 98 | 12 |
| AZO22 | Azores | MN716897 | 17 | - | - | - |
| AZO23 | Azores | MN716898 | 18 | - | - | - |
