# Supplementary material for: Genetic hypervariability of a Northeastern Atlantic venomous rockfish
Source: PeerJ. 2021 Jul 12;9:e11730. doi: 10.7717/peerj.11730 (PMC8280884; doi:10.7717/peerj.11730)
Supplement: Supplemental Information 2 [file peerj-09-11730-s002.docx]

| **Source of variation** | **df** | **Sum of squares** | **Variance component** | **% of variation** | ***F_ST_*** | ***p* value** |
| --- | --- | --- | --- | --- | --- | --- |
| *Mitochondrial control region* | | | | | | |
| Among sampling locations | 6 | 49.296 | 0.159 | 3.08 |  |  |
| Within sampling locations | 139 | 697.909 | 5.021 | 96.92 | 0.031 | 0.004 |
| *Nuclear S7* | | | | | | |
| Among sampling locations | 9 | 82.153 | 0.147 | 1.58 |  |  |
| Within sampling locations | 213 | 1953.975 | 9.174 | 98.42 | 0.016 | 0.005 |
