# Supplementary figures and images for: Genetic hypervariability of a Northeastern Atlantic venomous rockfish
Source: PeerJ. 2021 Jul 12;9:e11730. doi: 10.7717/peerj.11730 (PMC8280884; doi:10.7717/peerj.11730)

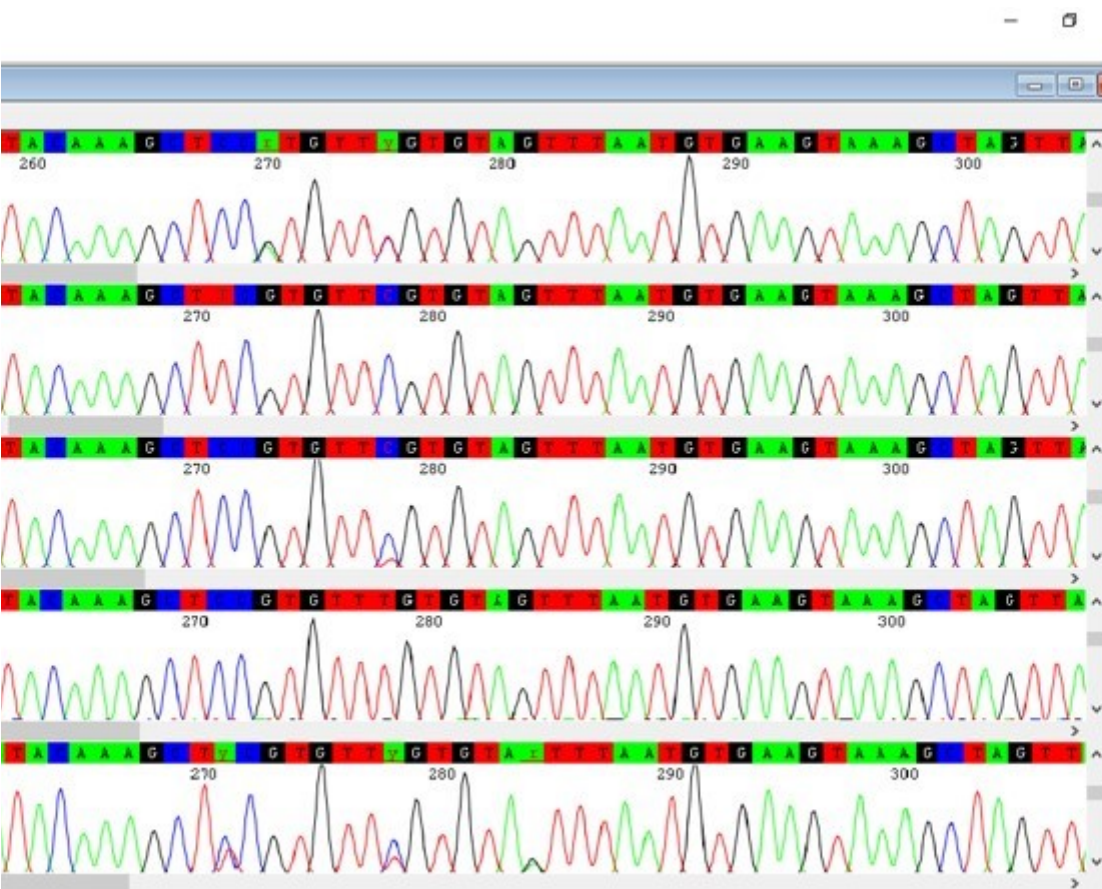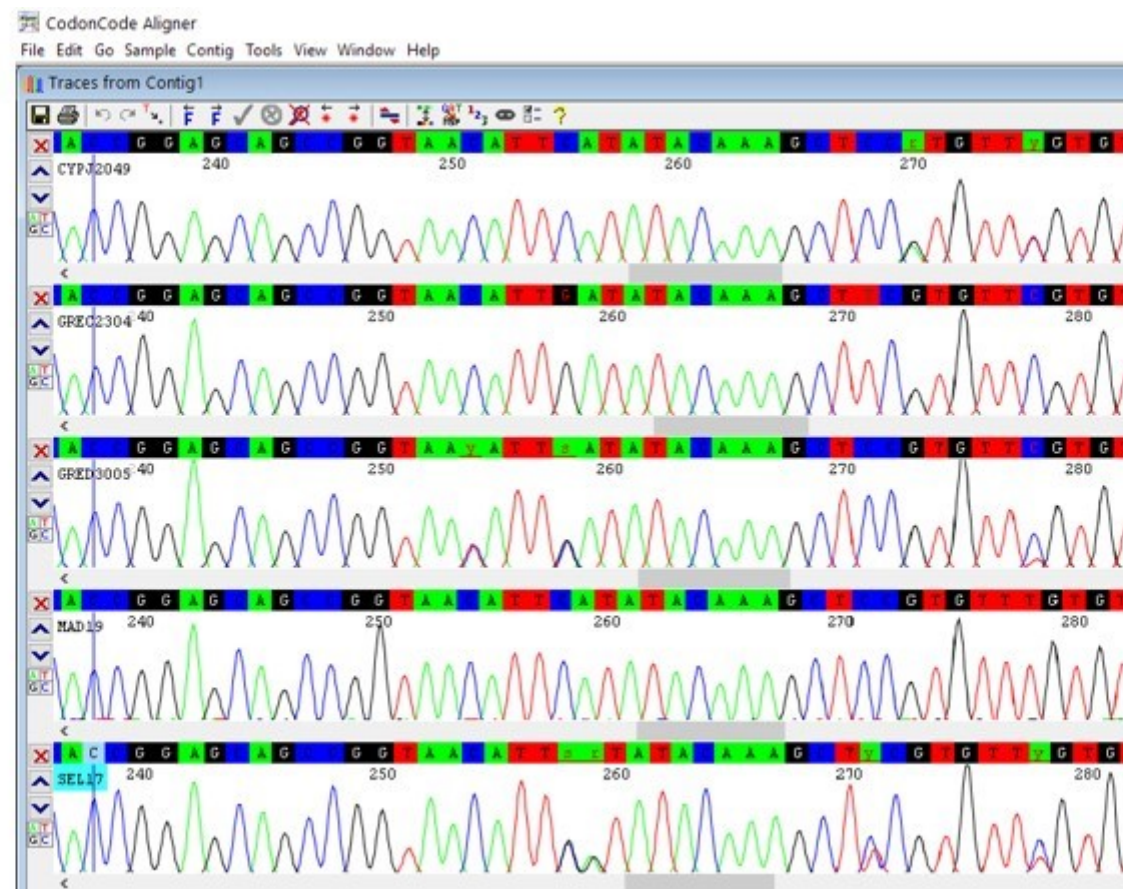

Supplement: Supplemental Information 3 [file peerj-09-11730-s003.pdf]
